# Pharmacological Insights of Ropivacaine and Clinical Applications: A Narrative Review

**DOI:** 10.7759/cureus.67565

**Published:** 2024-08-23

**Authors:** Shafaque Maqusood, Sheetal Madavi, Amol Bele, Sambit Dash, Dushyant Bawiskar

**Affiliations:** 1 Anaesthesiology, Jawaharlal Nehru Medical College, Datta Meghe Institute of Higher Education and Research, Wardha, IND; 2 Sports Medicine, Abhinav Bindra Targeting Performance, Bangalore, IND

**Keywords:** labor pain, post-operative, pain, local anesthesia, ropivacaine, bupivacaine

## Abstract

This review focuses on ropivacaine, a long-acting amide local anaesthetic, detailing its pharmacology and clinical applications. The article highlights its applications in providing analgesia, prolonging pain relief, and improving recovery outcomes in surgical settings. Ropivacaine is particularly effective for epidural labor analgesia in obstetrics, promoting stable hemodynamics and rapid onset when used with adjuvants. Its prolonged anesthetic effects reduce the need for postoperative opioids in peripheral nerve blocks. Intrathecal administration may enhance functional recovery and postoperative analgesia in various surgical procedures. While effective in treating acute pain, its role in chronic pain management remains unclear, indicating a need for further research. The review underscores the versatility and efficacy of ropivacaine in acute pain management and the importance of exploring its potential in chronic pain treatment.

## Introduction and background

The capability of a long-acting local anaesthetic to reversibly inhibit nerve impulses is vital for providing prolonged sensory or motor blockade, making it suitable for various surgical procedures [1,2]. Ropivacaine, a long-acting regional anaesthetic, bears structural similarities to bupivacaine. Unlike the racemic mixture of bupivacaine, ropivacaine is a pure S(-) enantiomer. This pattern aims to decrease potential toxicity while improving sensory and motor block profiles [3]. Ropivacaine is an amide with low lipid solubility and a high pKa (acid dissociation constant), selectively blocking A-delta and C nerve fibers involved in pain transmission more than A fibers responsible for motor function.

Ropivacaine is an amide local anaesthetic with an extended-lasting effect; it is designed as a single-optical isomer. This action is based on the reversible blockade of sodium channels in nerve fibers, like any other local anaesthetics, as its mechanism of action. Another long-acting regional anaesthetic of established efficacy is bupivacaine; however, all amide anaesthetics are known to cause cardiotoxic effects, particularly when concentration is high or when the drug is inadvertently administered intravascularly [4-6].

## Review

Pharmacodynamics

Ropivacaine, an amide local anaesthetic, inhibits nerve impulse conduction by reducing sodium ion permeability in nerve cell membranes. In vitro studies indicate that ropivacaine preferentially blocks A and C nerve fibers, which are responsible for pain transmission. By contrast, A-alpha nerve fibers are responsible for motor function due to their low lipid solubility and high pKa (8.2) [7]. This lower lipophilicity and stereoselective properties of ropivacaine result in a higher threshold for cardiotoxicity and central nervous system toxicity compared to bupivacaine in both animals and healthy individuals [8-10].

During intravenous (IV) infusion of local anaesthetics (10 mg/min of either ropivacaine or bupivacaine) in subjects, central nervous system (CNS) effects were observed before cardiotoxic symptoms, leading to the discontinuation of drug infusion [11,12]. Significant changes in heart contractility, conduction time, and QRS width were noted; however, the increase in QRS width with ropivacaine was significantly smaller than that observed with bupivacaine. In addition, ropivacaine has been shown to inhibit platelet aggregation in plasma at concentrations of 3.75 and 1.88 mg/mL (0.375% and 0.188%), which are similar to those that may occur during epidural infusion [13].

Pharmacokinetics

Ropivacaine exhibits linear and dose-proportional pharmacokinetics up to an 80 mg intravenous dose. When 150 mg is administered epidurally, it shows biphasic absorption with an initial half-life of 14 minutes, followed by a slower phase with a half-life of 4.2 hours [14]. Plasma concentration depends on the dosage, administration method, patient’s hemodynamic status, and vascularity at the administration site. Ropivacaine is 90-94% bound to plasma proteins, primarily α1-acid glycoprotein, and its Cmax (maximum concentration) for unbound drug remains below the CNS toxicity threshold (~0.6 mg/L) [15,16]. Ropivacaine crosses the placenta similarly to bupivacaine. In women undergoing cesarean sections, epidural administration of 20-28 mL of 0.5% or 0.75% ropivacaine results in mean maximum plasma concentrations of 1.1 to 1.6 mg/L. Due to higher α1-acid glycoprotein levels in maternal plasma, the total plasma concentration of ropivacaine is lower in fetal circulation than in maternal blood [17]. Ropivacaine is extensively metabolized in the liver, with only 1% excreted unchanged in urine. Its primary metabolite, 3-hydroxy-ropivacaine, is produced by CYP1A2 (cytochrome p450 family 1 subfamily A polypeptide 2), while CYP3A4 generates minor metabolites. Inhibitors of these enzymes, especially CYP1A2, can alter ropivacaine’s pharmacokinetics. For instance, coadministration with the CYP1A2 inhibitor fluvoxamine significantly increases ropivacaine plasma levels by 16% and delays its clearance [18].

Clinical applications

Use in Abdominal Surgical Anesthesia

Ropivacaine is a commonly used local anaesthetic in various surgical procedures, especially in abdominal surgeries. Studies have compared the efficacy of ropivacaine alone and in combination with other agents like fentanyl or dexmedetomidine for managing postoperative pain. Suryawanshi et al. compared the effectiveness of transversus abdominis plane (TAP) block using ropivacaine alone versus ropivacaine with fentanyl for postoperative analgesia in lower abdominal surgeries under general anaesthesia. Patients who received ropivacaine along with fentanyl had a higher quality of analgesia compared to those who received ropivacaine alone, indicating that the combination may be more effective in managing postoperative abdominal pain. Outcome measures used were the Visual Analog Scale, which assessed the severity of pain at multiple time points postoperatively. Another critical outcome measure was the time taken for patients to require rescue analgesia, specifically the administration of tramadol. These outcome measures collectively provided a comprehensive assessment of the effectiveness and safety of the TAP block techniques used in the study. The study found that patients receiving ropivacaine alone had a significantly earlier time to rescue analgesia compared to those receiving the combination of ropivacaine and fentanyl, indicating a difference in pain control effectiveness between the two groups [19]. A study by Byndoor et al. compared the efficacy and safety of levobupivacaine with ropivacaine in lower abdominal surgeries. Both levobupivacaine and ropivacaine demonstrated similar onset of sensory and motor blockade, along with comparable hemodynamic parameters [20].

In addition, the use of ropivacaine in TAP blocks has been found to provide a longer duration of analgesia compared to bupivacaine, enhancing postoperative pain relief. Ropivacaine 0.5% provided a significantly longer duration of analgesia compared to 0.25% bupivacaine when used in ultrasound-guided TAP block for postoperative pain relief in lower abdominal surgeries [21]. Furthermore, in the study by Elsharkawy et al., continuous preperitoneal infusion of ropivacaine has been shown to offer superior analgesic effects and improved pulmonary function compared to continuous epidural infusion in major abdominal and pelvic surgeries [22]. These findings collectively highlight the significant role of ropivacaine in enhancing pain management and recovery outcomes in general and abdominal surgeries.

Epidural Administration

Ropivacaine is a widely used local anaesthetic in various obstetric procedures. In the context of labor analgesia, studies have shown that adding clonidine as an adjuvant to ropivacaine results in a rapid onset of analgesia with a reduced required dose of ropivacaine without adverse neonatal effects. In a study by Karthik et al., the onset time of analgesia was significantly shortened when clonidine was added as an adjuvant to ropivacaine in epidural labor analgesia as compared with ropivacaine alone. The group that received ropivacaine with clonidine experienced a significantly quicker mean onset time of 17.7 ± 1.3 minutes compared to the group that received ropivacaine alone, which was 12.9 ± 1.3 minutes [23]. For cesarean sections, combining ropivacaine with dexmedetomidine prolongs sensory and motor block times, reduces chills, stress reactions, and inflammatory factors, and maintains stable hemodynamics, making it suitable for this procedure was found in a study done by Zhang et al. [24]. In addition, when Chen et al. compared different concentrations of ropivacaine for epidural analgesia during labor, both 0.1% and 0.068% ropivacaine provided similar pain relief and maternal temperature changes, indicating their comparable efficacy in this setting.

Furthermore, in epidural labor analgesia, 0.2% ropivacaine shows a faster onset of analgesia and shorter labor duration compared to 0.125% ropivacaine, with differences in required doses of ropivacaine and fentanyl between the two concentrations [25]. Ropivacaine offers significant benefits in pediatric patients undergoing various procedures. Studies have shown that ropivacaine when used in caudal blocks with or without adjuvants like fentanyl provides effective postoperative analgesia following infraumbilical surgeries in children aged three to eight years [26]. In addition, ropivacaine infiltration in the tonsillar fossa during tonsillectomy has demonstrated reduced postoperative pain and shorter operative times in pediatric patients [27]. Moreover, the utilization of ropivacaine in robotic-assisted surgery in children has been associated with decreased postoperative pain, shorter hospital stays, and improved surgical outcomes, highlighting its role in enhancing recovery and patient satisfaction [28].

Use in Peripheral Nerve Blocks

Ropivacaine plays a crucial role in peripheral nerve blocks due to its efficacy in providing prolonged anaesthesia and reducing the need for opioids postoperatively [29]. The duration of ropivacaine and dexmedetomidine blocks for the ulnar and posterior tibial nerves was compared in a study by Kettner SC, emphasizing the significance of dose-finding studies and safety information for peripheral nerve blocks. The findings showed that adding dexmedetomidine to ropivacaine increased the duration of the sensory block significantly, indicating that it may improve the effectiveness of nerve blocks [30]. Furthermore, continuous peripheral nerve blocks (CPNB) in children often utilize ropivacaine due to its favorable risk-benefit profile, especially when used at low concentrations and flow rates [31]. The addition of dexamethasone to ropivacaine has been found to further extend the duration of analgesia in single-shot inter-scalene blocks, with perineural and intravenous administration showing comparable efficacy in prolonging analgesia without adverse events [32]. Overall, ropivacaine remains a cornerstone in peripheral nerve blocks, especially when combined with appropriate adjuvants to optimize pain management and recovery outcomes. When used in combination with narcotics, such as in the context of regional anaesthesia for upper extremity surgeries, ropivacaine has demonstrated benefits in prolonging sensory anaesthesia without significantly affecting the duration of motor block [33]. This combination can lead to improved postoperative pain management, decreased narcotic consumption, and enhanced recovery outcomes, particularly in procedures like brachial plexus blocks for upper limb surgeries. Therefore, incorporating ropivacaine with narcotics in regional anaesthesia protocols can be a valuable strategy to optimize pain control and minimize opioid usage in surgical settings, aligning with the current efforts to combat opioid misuse and addiction, especially in adolescent populations undergoing procedures like anterior cruciate ligament reconstruction [34].

Intrathecal Administration

Roprivacaine administered intrathecally has been thoroughly investigated in a variety of settings. According to a study by Liu et al., intrathecal ropivacaine injection can alter the spinal cord's genomic structure and pain threshold, with genes primarily involved in inflammatory response pathways expressing differently [35,36]. A study by Huang et al. found that intrathecal continuous infusion of morphine and ropivacaine improved cancer patients' quality of life and effectively reduced their need for morphine. Compared to patients who received morphine alone, those who received ropivacaine had lower postoperative morphine requirements, higher Barthel Index scores, and lower Numerical Rating Scale (NRS) scores [37]. In a study led by Liu, 48 hours after surgery, patients who received ropivacaine showed improved active knee flexion and less need for analgesic pumps, indicating better pain management and functional outcomes [38]. Furthermore, compared to saline administration, intraarticular injection of ropivacaine into the knee joint following patellar fracture fixation has been demonstrated to improve postoperative analgesia, decrease local inflammatory response, and decrease the incidence of adverse reactions [38].

Chronic Pain Management

Ropivacaine has been extensively studied for its efficacy in pain management, particularly in chronic pain conditions. Albi-Feldzer et al. conducted a study [39]. After breast cancer surgery, ropivacaine wound infiltration significantly reduced postoperative pain, but it did not affect chronic pain at three, six, or 12 months after surgery. The complexity of managing chronic pain following breast cancer surgery is highlighted by the fact that, although ropivacaine wound infiltration demonstrated benefits in reducing immediate postoperative pain, it had no discernible effect on chronic pain outcomes over the long term [39]. Repetitive epidural administration of ropivacaine produced analgesic effects by possibly causing plastic changes in the nociceptive circuit, relieving mechanical allodynia and thermal hyperalgesia, according to research by Sato et al. on a rat model of neuropathic pain. According to the findings, ropivacaine administered repeatedly into the epidural space may cause plastic changes in the nociceptive circuit, which would then result in an analgesic effect. This suggests a possible mechanism for the clinically observed prolonged analgesic effect of local anaesthetics administered epidurally on chronic pain [40]. Significant evidence was presented by Raines et al. meta-analysis to support the clinical advantages of ropivacaine in lowering opioid use and successfully managing postoperative pain, underscoring the drug's potential as a substitute for conventional analgesic techniques. Ropivacaine continuous wound infusion is useful for postoperative pain management following a variety of surgical procedures because it has been demonstrated to produce clinically significant reductions in opioid use and pain outcomes. These findings collectively highlight the potential of ropivacaine in acute pain relief but suggest limited efficacy in managing chronic pain over extended periods postoperatively [41].

Intraperitoneal Ropivacaine

In a 2019 pilot study, Hayden et al. evaluated the effects of intraperitoneal ropivacaine on postoperative inflammation in patients undergoing surgery for ovarian cancer. The study randomized 40 patients to receive either intraperitoneal ropivacaine or saline, with 20 patients in each group. The results indicated that perioperative intraperitoneal ropivacaine did not significantly reduce the systemic inflammatory response associated with major abdominal surgery [42]. In a subsequent 2020 randomized controlled trial, Hayden et al. investigated whether intraperitoneal ropivacaine could reduce the time interval to the initiation of chemotherapy following surgery for advanced ovarian cancer. Of the 88 women assessed for eligibility, 58 were included in the study. The authors concluded that intraperitoneal ropivacaine administered during surgery and for 72 hours postoperatively after cytoreductive surgery is safe and effectively reduces the time to initiation of chemotherapy. They also suggested that further studies with larger sample sizes are needed to confirm these findings [43]. In a 2013 placebo-controlled trial, Ingelmo et al. examined the effect of intraperitoneal nebulization of ropivacaine on pain control following laparoscopic cholecystectomy. Patients were randomized into three groups: one received intraperitoneal nebulization of ropivacaine 1% (3 ml) before surgical dissection and normal saline (3 ml) at the end of surgery (preoperative nebulization group); another received normal saline (3 ml) before surgical dissection and ropivacaine 1% (3 ml) at the end of surgery (postoperative nebulization group); and the placebo group received normal saline (3 ml) both before and after surgery. The study found that ropivacaine nebulization, whether administered before or after surgery, significantly reduced postoperative pain, including referred shoulder pain, decreased morphine requirements, and facilitated earlier patient mobility following laparoscopic cholecystectomy [44].

Preventing Cancer Recurrence

Zhang et al. did a meta-analysis in 2021. A total of three RCTs and 34 cohort studies were included in the study. Authors found that overall survival and recurrence-free survival were similar between regional and general anaesthesia in late-stage cancers [45]. Wang et al. did a study in 2021, which aimed to investigate the effects of ropivacaine on the proliferation, cell cycle, and apoptosis of colon cancer cells. This study showed that under the action of ropivacaine, the expression of β1 integrin (ITGB1) in HCT116 and subcutaneous colorectal cancer xenograft tumor model (SW620) cells was significantly reduced, and ropivacaine can inhibit the proliferation, migration, and invasion of colorectal cancer cells. In addition, we confirmed for the first time that ropivacaine regulates the biological function of colon cancer cells through ITGB1 [46]. A study by Jia in 2023 aimed to find out if ropivacaine inhibits the malignant behavior of lung cancer cells by regulating retinoblastoma-binding protein 4 and found that ropivacaine suppresses lung cancer cell malignancy by downregulating RB binding protein 4 expression, which may help clarify the mechanisms underlying the antitumor effects of ropivacaine [47].

Adverse Effects

Common systemic side effects of ropivacaine include neuroleptic malignant-like syndrome, which can present with symptoms such as confusion, agitation, and fever [41]. In addition, ropivacaine has been noted for its lesser cardiotoxic effects compared to other local anaesthetics like bupivacaine, making it a safer choice for postoperative pain management. Furthermore, the side effects of drug annual reviews highlight that ropivacaine, like other local anaesthetics, can also be linked to allergic reactions, hemodynamic instability, and neurological deficits, emphasizing the importance of proper monitoring and management when using this medication [48,49]. 

Rare allergic reactions associated with ropivacaine, an amide-type local anaesthetic, include severe anaphylactic reactions leading to cardiovascular collapse [50]. Allergic reactions to amide local anaesthetics like ropivacaine are infrequent compared to ester-type local anaesthetics. IgE antibodies mediate these reactions and are extremely rare, accounting for less than 1% of all adverse reactions to local anaesthetics. In pediatric patients, the confirmed incidence of local anaesthetic allergy is low, with only one out of 73 patients showing a positive response to mepivacaine during diagnostic testing. Monitoring and diagnosing such rare allergic reactions are crucial, especially in cases where severe or life-threatening reactions occur, emphasizing the importance of selecting safe local anaesthetics based on individual patient histories and allergy testing [51].

Discussion

Research conducted on ropivacaine exhibits its versatility in its utilization across these clinical operations and diverse anaesthetic methods. In surgical anaesthesia, especially in cases of abdominal surgeries, ropivacaine has proved promising as compared to bupivacaine and other similar drugs. Some studies have also demonstrated its efficacy and postsurgical analgesia when used with adjuvants such as fentanyl and dexmedetomidine. An improvement in the quality of analgesia was depicted in a study by Suryawanshi et al. The other studies exemplified by Byndoor et al. and Elsharkawy et al. have also shown great efficacy and safety in ropivacaine-fentanyl with other local anaesthetics such as levobupivacaine and continuous preperitoneal infusion. This added duration of analgesia, as compared to other agents like bupivacaine, leads to the strong use of ropivacaine in postoperative pain, especially in transverse abdominis plane (TAP) blocks [19-21].

Regarding obstetric applications, ropivacaine epidural use is backed by findings demonstrating the effectiveness of the anaesthetic during childbirth and cesarean section. Karthik et al. ’s outcome on the onset of analgesia when clonidine is used as an adjuvant and Zhang et al.’s study on the stability of hemodynamics and reduced stress reactions with the use of dexmedetomidine give hope for improving epidural labor analgesia. Chen et al.'s study and other studies also support the idea of the analgesic equivalency between different ropivacaine concentrations for labor pain relief while stressing concentration modulation in clinical practice [23-25].

Peripheral nerve blocks are facilitated by the long-lasting anaesthetic effect of ropivacaine and reduced postoperative opioid use. Research such as those carried out by Kettner SC and other works on topics like the use of adjuvants, including dexmedetomidine and dexamethasone, underscores the duration of ropivacaine-based nerve blocks and increased efficacy. While using ropivacaine through the intrathecal route, there are also observed positive results in different types of surgical procedures, specifically for the improvement of postoperative pain management and the patient’s mobilization, with references to the studies of Liu et al. and Chen et al. [30-39].

However, more about ropivacaine in chronic pain is still ambiguous. Accomplishments involving its utilization have been previously established in the alleviation of acute surgical pain; nevertheless, research works such as that of Aline Albi-Feldzer et al. suggest suboptimal effectiveness for chronic pain after surgery. On the other hand, repeated epidural administration implies the potential to activate plastic changes in the nociceptive circuit, which may be useful in chronic pain disorders, as demonstrated by Sato et al. Lastly, the meta-analysis by Raines et al. reveals ropivacaine’s clinical benefits in minimizing the need for opioids and addressing post-surgical pain. However, ropivacaine seems less efficient for chronic pain conditions in patients operated on. Such perceptions, served in harmony, place ropivacaine’s versatile applicability as credible and efficacious for acute pain instances while revealing the necessity of additional study about chronic pain uses [39-41].

Recent studies have explored the potential anticancer effects of ropivacaine, adding a new dimension to its clinical application. For instance, Zhang et al.'s 2021 meta-analysis, which included three randomized controlled trials (RCTs) and 34 cohort studies, revealed no significant difference in overall survival and recurrence-free survival between regional and general anaesthesia in late-stage cancers. By contrast, experimental studies have demonstrated ropivacaine's ability to inhibit cancer cell proliferation and metastasis. Wang et al. (2021) showed that ropivacaine reduces the expression of β1 integrin (ITGB1), thereby inhibiting the proliferation, migration, and invasion of colorectal cancer cells. Moreover, Jia et al. (2023) found that ropivacaine suppresses lung cancer cell malignancy by downregulating retinoblastoma-binding protein 4 (RBBP4), suggesting a potential role for ropivacaine in targeting specific molecular pathways involved in tumor progression [42-47].

## Conclusions

Ropivacaine has shown to be a very useful and effective local anaesthetic, offering notable advantages in applications such as peripheral nerve blocks, obstetrics, and surgery. Its capacity to deliver sustained analgesia, especially in conjunction with adjuvants such as clonidine, dexmedetomidine, and fentanyl, improves patient recovery and postoperative pain management by stabilizing hemodynamic parameters and lowering the need for opioids. Although its effectiveness in treating acute pain is well-established, more studies are required to fully investigate its potential in managing chronic pain and to maximize its application in a range of clinical contexts. Ropivacaine's advantageous safety profile, effectiveness, and versatility make it a useful tool in modern anaesthesia practice, greatly enhancing patient outcomes and satisfaction.
